# Regulation of Myeloid Dendritic Cells by Synthetic and Natural Compounds for the Treatment of Rheumatoid Arthritis

**DOI:** 10.3390/ijms24010238

**Published:** 2022-12-23

**Authors:** Hira Umbreen, Xiang Zhang, Kuo-Tung Tang, Chi-Chien Lin

**Affiliations:** 1 Rong Hsing Research Center for Translational Medicine, National Chung Hsing University, Taichung 402, Taiwan; 2 Department of Molecular Medicine and Surgery, Karolinska Institute, 171 76 Stockholm, Sweden; 3 Division of Allergy, Immunology, and Rheumatology, Taichung Veterans General Hospital, Taichung 407, Taiwan; 4 Faculty of Medicine, National Yang Ming Chiao Tung University, Taipei 112, Taiwan; 5 Ph.D. Program in Translational Medicine, National Chung Hsing University, Taichung 402, Taiwan; 6 Department of Medical Research, Taichung Veterans General Hospital, Taichung 407, Taiwan; 7 Institute of Biomedical Science, The iEGG and Animal Biotechnology Center, National Chung-Hsing University, Taichung 402, Taiwan; 8 Department of Medical Research, China Medical University Hospital, Taichung 404, Taiwan; 9 Department of Pharmacology, College of Medicine, Kaohsiung Medical University, Kaohsiung 807, Taiwan

**Keywords:** dendritic cell, drug, natural product, plant, rheumatoid arthritis, synthetic compounds, treatment

## Abstract

Different subsets of dendritic cells (DCs) participate in the development of rheumatoid arthritis (RA). In particular, myeloid DCs play a key role in the generation of autoreactive T and B cells. Herein, we undertook a literature review on those synthetic and natural compounds that have therapeutic efficacy/potential for RA and act through the regulation of myeloid DCs. Most of these compounds inhibit both the maturation of DCs and their secretion of inflammatory cytokines and, subsequently, alter the downstream T-cell response (suppression of Th1 and Th17 responses while expanding the Treg response). The majority of the synthetic compounds are approved for the treatment of patients with RA, which is consistent with the importance of DCs in the pathogenesis of RA. All of the natural compounds are derived from plants. Their DC-modulating effect has been demonstrated both in vitro and in vivo. In addition, these natural products ameliorate arthritis in rodents and are potential therapeutics for human RA.

## 1. Introduction

Rheumatoid arthritis (RA) is a polyarticular inflammatory illness, which typically affects the hands and feet [1]. The prevalence of RA is 1% worldwide, with a female predominance [2,3]. The average age at the onset of RA is between 40 and 50 years, and the male:female ratio is from 1:2 to 1:3 [4]. A variety of joints are affected by RA, such as those of the hand, foot, wrist, knee, elbow, and ankle [5]. Patients with RA typically present with joint swelling and pain, and this may progress to notable functional impairment, negatively impacting the physical and mental well-being of the patients [6]. Predisposing factors for RA are genetic predisposition, immunological dysregulation (loss of immune self-tolerance and generation of autoantibodies), sex hormones (estrogen), infection, and environment [5,7]. The pathological manifestations of RA include persistent inflammation in synovial joints, pannus formation, progressive erosion of periarticular bone, and severe destruction of joint structure [8]. Furthermore, systemic inflammation is harmful to a variety of organ systems, including the heart, kidney, lung, vascular system, and neurological system [9,10]. If RA is not well treated with systemic therapies, the damage to the joints and other organs will culminate in significant functional impairment and even death [11].

The etiology of RA remains elusive. Currently, several factors are linked to its pathogenesis [12]. Environmental factors such as smoking contribute to the development and severity of RA [13]. In particular, smoking interacts with the genetically determined shared epitope alleles of human leukocyte antigen (HLA)-DR to increase the risk of developing RA [14]. Periodontitis and its pathogens, Porphyromonas gingivalis and Aggregatibacter actinomycetemcomitans, are involved in the breakdown of immune tolerance in RA [15,16]. Dysbiosis of the gut microbiota leads to chronic inflammation, which facilitates the generation of RA [17]. The dysregulation of the immune system, e.g., the breakthrough of immune tolerance to self-antigens, also plays a key role [3]. Smoking induces lung inflammation and facilitates the local citrullination of proteins. The resultant neoepitope could stimulate the immune system to produce autoantibodies [18]. In line with that, circulating autoantibodies, such as rheumatoid factors (RFs) and anti-citrullinated protein antibodies (ACPAs), are detected before the onset of RA symptoms, reflecting the gradual and progressive nature of the underlying autoimmune process [19,20]. Complex interactions between immune cells and synovial tissue result in progressive bone erosion [19,20]. The proliferating T and B lymphocytes, monocytes, and neutrophils together with synovial fibroblasts contribute to joint inflammation [21]. Such inflammation causes the synovium to thicken and results in the formation of pannus, an aberrant tissue that invades nearby articular structures. In addition, pro-inflammatory chemokines and cytokines, as well as matrix metalloproteinases, are produced by the pannus tissue, contributing further to cartilage and bone degradation [21,22].

In the joint microenvironment, inflammation starts first in the synovium and progresses to the interstitial zone covered by intimal synovial fibroblasts and macrophage-like synoviocytes (MLSs) [4,23,24]. The interstitial zone is made up of infiltrating macrophages, mast cells, T cells, B cells, and synovial fibroblasts. B cells transform into plasma cells, which produce RFs and ACPAs [25]. Macrophages and synovial fibroblasts produce an abundance of pro-inflammatory cytokines, such as interleukin (IL)-1, IL-6, and tumor necrosis factor (TNF)-α [26]. Moreover, synovial cells release TNF-α and tissue-degrading matrix metalloproteinases (MMPs), and they in turn trigger the differentiation and proliferation of osteoclasts [4]. Hence, in a vicious cycle, additional macrophages, fibroblasts, and lymphocytes are recruited and activated, generating an exaggerated inflammatory response [26]. Consistent with this picture, high levels of IL-1, IL-6, IL-8, IL-17, TNF-α, MMPs, and granulocyte colony-stimulating factor (G-CSF) have been found in the synovium and synovial fluid of joints affected by RA. Furthermore, vascular endothelial growth factor (VEGF) and the receptor activator of nuclear factor-κB ligand (RANKL) can regulate osteoclasts and cause bone degradation. VEGF also promotes angiogenesis and the recruitment of additional inflammatory leukocytes, further promoting joint inflammation [27].

Dendritic cells (DCs) are crucial in the elicitation of the inflammatory response. Numerous studies have explored the potential of DC modulation in the treatment of patients with RA. Notably, several synthetic and natural compounds have been implicated based on the treatment strategy. Here, we summarize recent developments in regard to DC-modulating compounds in RA treatment. Our literature review is expected to provide evidence on new therapeutics for RA.

## 2. Dendritic Cells

DCs are cells that present antigens by means of their specialized function. DCs participate in the first-line innate immune response and elicit adaptive immune reactions. These cells collect foreign antigens and present to the immune system, and they are key to the downstream inflammatory response. DCs are sentinels for the immune system and resident in most organs. These cells are characterized by their distinct and unique “tree-like” dendritic shape, in addition to high levels of expression of major histocompatibility complex (MHC) class II molecules [28]. DCs are a heterogeneous population in terms of phenotypic and transcriptional profile. Such heterogeneity is shown in the development stage, maturation status, and tissue context. DC subsets have diverse functions [29,30]: conventional DC1 (cDC1) is capable of cross-presentation of antigen to CD8+ T cells; cDC2 and blood monocyte-derived DCs (moDCs) can initiate CD4+ T-cell responses (e.g., Th1, Th2, and Th17) dependent on the inflammatory signals they receive; plasmacytoid DCs (pDCs) rapidly produce type I interferon (IFN) once they encounter danger signals. In particular, myeloid DCs can prime the downstream T-cell response and promote the activation, expansion, and differentiation of CD4+ effector T cells. Infectious agents or endogenous danger signals (e.g., extracellular DNA and RNA) interact with DCs through pattern recognition receptors (e.g., Toll-like receptors) that are expressed on the surface of DCs. In turn, DCs release cytokines and growth factors that pivot the downstream T- and B-cell immune responses. In addition, DCs interact with other immune cells, such as natural killer and innate lymphoid cells (ILCs), during the immune response [31,32,33,34].

DCs are categorized into two functional states: mature and immature. Mature and immature DCs differ in a number of aspects. The ability of mature DCs to secrete a myriad of cytokines and pivot the activation of different lineages of antigen-specific T lymphocytes (e.g., Th1, Th2, Th17, and Treg cells) in secondary lymphoid organs is the most important difference [35,36]. Pathogen-associated molecular patterns (PAMPs), or damage-associated molecular patterns (DAMPs), and several inflammatory cytokines promote DC maturation [37,38]. During the maturation process, DCs express CD80, CD86, and MHC-II on the surface and downregulate their phagocytic capacity, thereby facilitating their interaction with T cells [39]. It should be noted that researchers have already examined the therapeutic potential of DC-based immunotherapy (e.g., regulating DC maturation) in malignant, infectious, and autoimmune diseases [40,41]. For example, the administration of growth factor FLT3 ligand followed by intratumoral poly I:C injection expands tumor DCs and inhibits melanoma growth in mice [42]. The pulmonary delivery of activated DCs, which are primed by the Mycobacteria tuberculosis antigen, could enhance vaccine-induced protection and limit Mycobacteria growth in vaccinated mice [43]. Self-peptides without adjuvants, delivered by antibodies targeting the c-type lectin receptor DEC205 on DCs, lead to the generation of tolerance and effectively ameliorate autoimmune diseases, such as experimental autoimmune encephalomyelitis, diabetes, and, colitis, in mice [41].

Our present review focuses on myeloid DCs in RA and, for simplicity, refers to myeloid DCs as DCs in the following text.

## 3. The Role of Dendritic Cells in Pathogenesis of RA

**Figure 1:**
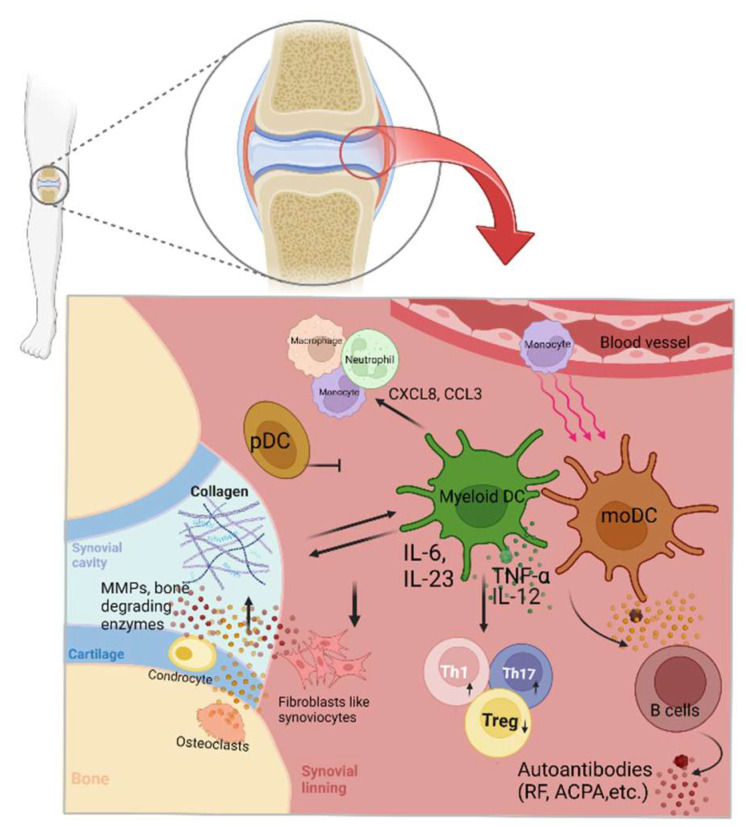
An illustration of the pathogenic role of dendritic cells in rheumatoid arthritis. ACPA, anti-citrullinated protein antibody; IL, interleukin; MMP, metalloproteinase; moDC, monocyte-derived dendritic cell; pDC, plasmacytoid dendritic cells; RF, rheumatoid factor; TNF, tumor necrosis factor.

DCs are key to the balance between immune activation and tolerance [44]. Dysregulated DCs play a crucial role in autoimmunity. In the absence of DCs, a fatal autoimmune phenomenon develops in mice [45,46]. On the other hand, antigen presentation by DCs and the formation of an immunological synapse with T cells [47] require two activating signals, which lead to T-cell activation [48,49]. The co-stimulatory molecules include lymphocyte function-associated antigen 1 (LFA-1)/intercellular adhesion molecule 1 (ICAM-1), CD2/LFA-3, and CD28/B7-1 [50,51]. Some fusion proteins and monoclonal antibodies targeting these co-stimulatory molecules have been developed to treat autoimmune diseases, including RA [52]. These biological agents, including cytotoxic T-lymphocyte-associated protein 4 (CTLA-4)-Ig, LFA-3-Ig, and anti-CD3 monoclonal antibody, could prevent the successful engagement of DCs by T cells, achieving significant therapeutic efficacy [53,54]. All DC subsets are known to infiltrate joints in patients with RA and even appear in lymph nodes before arthritis development (Figure 1) [55]. In addition, monocyte-derived DCs from patients with RA, when compared with healthy controls, secrete more pro-inflammatory chemokines (CXCL8 and CCL3) and cytokines (IL-6 and IL-23); skew the T-cell differentiation toward the Th17 lineage at the expense of regulatory T (Treg) cells; and attract more macrophages, neutrophils, and monocytes. Meanwhile, synovial DCs in patients with RA express activation markers, stimulate T cell proliferation, and attract effector T cells with greater chemokine (CCL17, CXCL9, and CXCL10) secretions. An earlier study in mice reported that arthritis is induced by intra-articular injection of collagen-specific DCs [46]. Myeloid DCs likely contribute to the initiation and perpetuation of RA [56]. First, DCs prime and activate T cells, leading to local and systemic inflammation in RA. Second, DCs secrete a myriad of inflammatory mediators that drive the activation of innate immune cells [46,57], and ectopic lymphoid structures appear in joints affected by RA [58]. Of note, collagen II has been shown to induce DC maturation, and mature DCs, in turn, induce collagen degradation in the joint tissue. Such a vicious cycle facilitates joint destruction [59]. In contrast to the contributory role of myeloid DCs, pDCs appear to inhibit the generation of RA. Depleting pDCs exacerbate arthritis and the inflammatory response in mice [60].

## 4. DC-Targeting Strategies for the Treatment of RA

Due to the substantial contribution of DCs to RA pathogenesis, various DC-modulating therapies have been developed. Current biological therapies of RA ameliorate the disease by targeting downstream products of DCs [44], such as TNF-α, IL-1 (α and β), and IL-6 [61]. Emerging RA therapies exploit the tolerogenic capacity of DCs. Tolerogenic DCs can be generated from myeloid precursors ex vivo, loaded with antigens and manipulated to suppress the autoimmune response in vivo. Such DCs induce T-cell anergy and/or regulatory T cells [62,63]. To generate tolerogenic DCs, many researchers have pulsed bone-marrow-derived dendritic cells (BMDCs) and human moDCs with rosiglitazone, vasoactive intestinal peptide, dexamethasone/vitamin D3, nuclear factor κB (NF-κB) inhibitor, or nuclear receptors REV-ERB inhibitor [64,65,66,67,68]. In the mouse model and patients with RA, re-infusion of tolerogenic DCs alleviate their disease symptoms. Larger clinical trials to validate these tolerance-inducing approaches are required before their application in clinical practice [69].

## 5. Compounds with Therapeutic Efficacy/Potential for RA through DC Regulation

Recently, several compounds have been shown to regulate DCs and, therefore, ameliorate RA symptoms in animal models and humans. We herein summarized these compounds and described their DC-modulating effects and therapeutic efficacy in RA.

### 5.1. Synthetic Compounds

**Figure 2:**
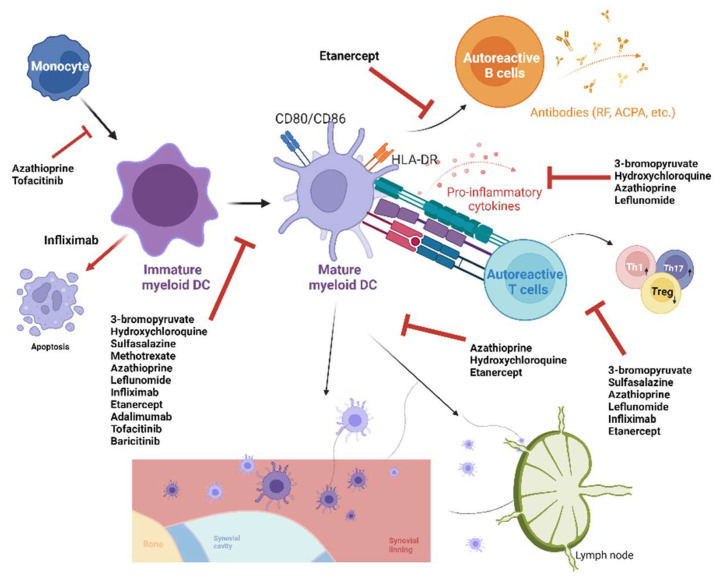
Synthetic compounds with dendritic cell (DC)-modulating effects and therapeutic efficacy in rheumatoid arthritis. ACPA, anti-citrullinated protein antibody; HLA, human leukocyte antigen; RF, rheumatoid factor.

We present a variety of synthetic compounds with potential therapeutic efficacy for RA through the regulation of DCs [70,71,72,73,74,75,76,77,78,79,80,81,82,83]. Most of these compounds, with the exception of 3-bromopyruvate, are currently approved for clinical use for patients with RA (Table 1). Nevertheless, the development of these medications did not depend on their pharmacological effects on DCs. Rather, the beneficial effects relevant to DCs were found after their clinical use. We noted that these approved medications, including conventional, biological, and targeted synthetic disease-modifying antirheumatic drugs (DMARDs), could suppress DC function and downstream T-cell response based on evidence from in vitro experiments. These findings further support the importance of DCs in the pathogenesis of RA. Mechanistically, conventional DMARDs exert their inhibitory effects on DCs through mechanisms other than their known pharmacological actions (Figure 2). These conventional DMARDs have a variety of actions, such as downregulating the expression of TLR9 (hydroxychloroquine) [71], suppressing the formation of reactive oxygen species (ROS) and Na+/H+ exchanger activity (involved in the regulation of cytosolic pH and migration) (azathioprine) [75], and suppressing nuclear factor-κB (NF-κB) activation (leflunomide and sulfasalazine) [72,76] in DCs. Biological DMARDs primarily exert their effect through the inhibition of specific cytokines. Inflammatory cytokines, such as TNF-α and IFN-α, could stimulate DC maturation [84]. This may partly explain their in vivo inhibitory effects on DCs as a consequence of dampened inflammation after the administration of biological DMARDs. The inhibitory effect of these biological DMARDs on DC function in vitro is presumably mediated through either the autocrine pathway or reverse signaling [77]. For targeted synthetic DMARDs, such as Janus kinase (JAK) inhibitors, intracellular signaling of multiple cytokines can be inhibited. Particularly, type I IFN signaling through JAK1 is suppressed, and DC function is inhibited in a way similar to that described above [83].

### 5.2. Natural Compounds

**Figure 3:**
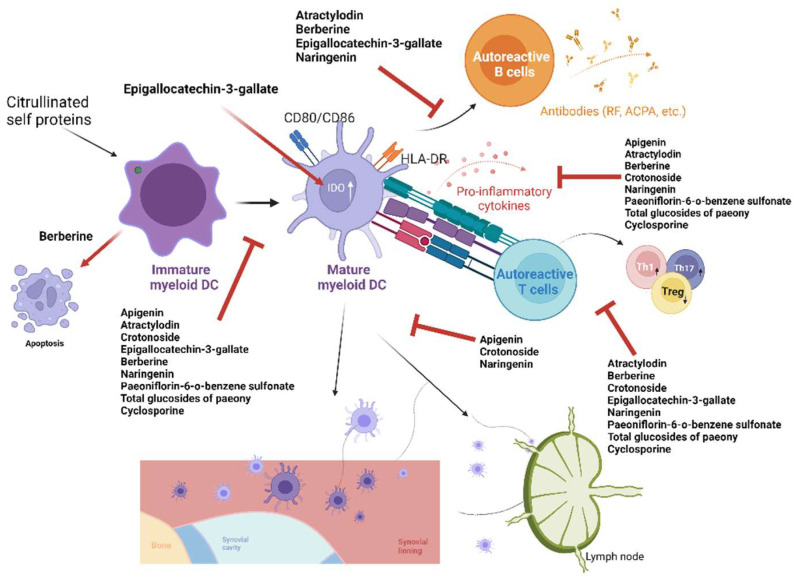
Natural compounds with dendritic cell (DC)-modulating effects and therapeutic efficacy in rheumatoid arthritis. ACPA, anti-citrullinated protein antibody; HLA, human leukocyte antigen; RF, rheumatoid factor.

Natural products are a rich source of potential therapies for various diseases. For instance, cyclosporine, an immunosuppressant often used in patients with transplantation and autoimmune diseases, including RA, is initially extracted from a fungus [85]. Pilocarpine, a parasympathetic agonist for the treatment of dry mouth, is found in a Brazilian plant [86]. In addition, the side effects of these natural products are presumably mild, particularly if they are extracted from herbs or foods, which have been consumed by humans for thousands of years. Herein, we summarized the natural products used as potential therapeutic agents for RA through the regulation of DCs (Table 2) [87,88,89,90,91,92,93,94,95]. All these compounds are extracted from plants. Only cyclosporine has been approved for RA treatment. These compounds suppress the maturation of DCs and, in turn, their ability to secrete inflammatory cytokines, such as TNF-α, IL-6, etc.; to stimulate T cell proliferation; to stimulate B cells to produce antigen-specific antibodies; and to pivot T helper cells toward the Th1 and Th17 responses (Figure 3). Such effects are reported in both in vitro and in vivo experiments. In rodent models, these compounds ameliorate experimental arthritis. In terms of an underlying mechanism, these compounds inhibit mitogen-activated protein kinase (MAPK) and NF-κB signaling (atractylodin and naringenin) [88,93], downregulate chemokine receptor 4 (CXCR4) (apigenin) [87], and induce indoleamine-2,3-dioxygenase (IDO) expression (epigallocatechin-3-gallate) [92] in DCs. The mechanisms of these compounds appear to be different from those of synthetic compounds as mentioned above.

## 6. Limitations

Our review is limited in several respects. First, some of these compounds, while exerting inhibitory effects on DC function, also affect other immune cells. We could not determine whether the suppression of DCs primarily leads to the alleviation of arthritis symptoms, although previous animal and human studies demonstrated the therapeutic efficacy of tolerogenic DCs in RA. Second, the toxicity of the natural compounds is not fully addressed in these studies, though they show no cytotoxicity in DCs. For instance, adverse effects on other organ systems, such as the liver and kidneys, should be further investigated before clinical use. Third, there is limited examination of the therapeutic efficacy of the natural compounds in primates and in humans. Nevertheless, it is worthwhile at this stage to summarize the relevant research findings of these compounds and find novel agents with therapeutic potential.

## 7. Conclusions

DCs represent the link between the systems of innate and adaptive immunities, and they are critical in the aberrant immune response in patients with RA. In line with this, most medications approved for RA treatment have been shown to suppress DC function and downstream T-cell response. Furthermore, several natural compounds, principally derived from plants, also inhibit the maturation and secretion of inflammatory cytokines in DCs, and downstream T- and B-cell responses, as demonstrated by in vitro and in vivo experiments. Furthermore, in the mouse model, these natural compounds are therapeutically effective against RA symptoms. They have promising therapeutic potential for RA treatment. Nevertheless, their absorption, distribution, metabolism, excretion, and potential toxicity to major organ systems (the cardiovascular, lung, liver, kidney, hematological, and reproductive systems) in humans should be determined. Genotoxicity and carcinogenicity studies should be undertaken. Moreover, these natural compounds may need to undergo modification through either chemical synthesis or biosynthetic engineering to enhance their efficacy, obtain a better pharmacokinetic profile, and reduce their toxicity [96]. Further human studies and even randomized controlled trials should then be implemented to explore their clinical efficacy and toxicity.

## Figures and Tables

**Table 1:** Synthetic compounds with therapeutic efficacy/potential for rheumatoid arthritis and their pharmacological effects on dendritic cells (DCs).

Synthetic Compounds	In Vitro Effect on DCs	In Vivo/Ex Vivo Effect on DCs	Reference Number
3-bromopyruvate	Suppressed maturation and secretion of inflammatory cytokines of BMDCs	Suppressed downstream Th17 response and increased Treg response	[70]
Conventional DMARDs			
Hydroxychloroquine	Suppressed maturation, migration, secretion of inflammatory cytokines in peripheral blood DCs, and maturation and migration of BMDCs	Decreased number of DCs and their maturation in lymph node of mice	[71]
Sulfasalazine	Suppressed maturation of moDCs and downstream T-cell proliferation	N.A.	[72]
Methotrexate	N.A.	Suppressed maturation of lymph node and splenic DCs in mice	[73]
Azathioprine	Suppressed differentiation, activation, migration, and secretion of inflammatory cytokines of moDCs and downstream T-cell proliferation	N.A.	[74,75]
Leflunomide	Suppressed maturation and secretion of inflammatory cytokines of moDCs and downstream T-cell proliferation	N.A.	[76]
Biologic DMARDs			
Infliximab (an anti-TNF-α monoclonal antibody)	Increased moDC apoptosis, suppressed maturation of mDCs, and downstream T-cell proliferation and Th1 response while increasing Treg response	Increased blood DCs	[77,78]
Etanercept (a TNF-α receptor fusion protein)	Suppressed maturation and migration of BMDCs	Reduced number and suppressed maturation and migration of lymph node DCs and downstream T- and B-cell proliferation in mice	[79]
Adalimumab (an anti-TNF-α monoclonal antibody)	Suppressed maturation of moDCs	N.A.	[80]
Tocilizumab (an anti-IL-6 receptor antibody)	N.A.	Decreased blood DCs	[81]
Targeted synthetic DMARDs			
Tofacitinib	Suppressed differentiation, activation, and maturation of moDCs	N.A.	[82,83]
Baricitinib	Suppressed maturation of moDCs	N.A.	[83]

BMDCs: bone-marrow-derived dendritic cells; DMARDs: disease-modifying antirheumatic drugs; IL: interleukin; moDCs: monocyte-derived dendritic cells; N.A.: not available; TNF: tumor necrosis factor.

**Table 2:** Natural compounds with therapeutic potential for rheumatoid arthritis (RA) and their pharmacological effects on dendritic cells (DCs).

Compounds	Source	Animal Models of RA	In Vitro Effect on DCs	In Vivo Effect on DCs	Reference Number
Apigenin	*Matricaria chamomilla*	CIA in mice	Suppressed maturation, migration, and secretion of inflammatory cytokines in BMDCs	Suppressed number and maturation of lymph node DCs	[87]
Atractylodin	*Atractylodis rhizoma*	CIA in mice	Suppressed maturation and secretion of pro-inflammatory cytokines and nitric oxide of BMDCs, and downstream T-cell proliferation	Suppressed splenic DC maturation and downstream anti-CII antibody production and splenic Th1/Th17 responses	[88]
Berberine	*Berberis* spp. and *Coptis* spp.	CIA in mice	Induced apoptosis and suppressed maturation of BMDCs	Induced apoptosis and suppressed maturation of splenic and lymph node DCs with suppressed anti-CII antibody production, and downstream collagen-specific T-cell proliferation and Th1 and Th17 responses	[89]
Crotonoside	*Croton tiglium*	CIA in mice	Suppressed differentiation, maturation, production of inflammatory cytokines in BMDCs, and downstream T-cell activation and Th1/Th17 responses	Suppressed DC infiltration of joint, splenic DC maturation, and downstream Th1 and Th17 responses	[90]
Cyclosporine	*Tolypocladium inflatum*	N.A.	Suppressed maturation and secretion of inflammatory cytokines of BMDCs and downstream T-cell response	N.A.	[91]
Epigallocatechin-3-gallate	*Camellia sinensis* (green tea)	CIA in mice	Increased IDO expression in splenic DCs and downstream Treg response	Increased IDO-producing lymph node DCs, downstream Treg response, and suppressed anti-CII antibody production	[92]
Naringenin	*Citrus* spp.	CIA in mice	Suppressed maturation, migration, and secretion of inflammatory cytokines in BMDCs and downstream T-cell proliferation	Suppressed anti-CII antibody production and downstream Th1 and Th17 responses in the spleen	[93]
Paeoniflorin-6′-O-benzene sulfonate (CP-25)	Paeonia	Adjuvant-induced arthritis in rats	Suppressed maturation and secretion of inflammatory cytokines of BMDCs and downstream T-cell proliferation	Suppressed maturation of peripheral blood DCs in patients	[94]
Total glucosides of paeony	*Paeonia* spp.	CIA in mice	Suppressed maturation, production of inflammatory cytokines of BMDCs, and downstream Th1/Th17 responses	Suppressed splenic DC maturation, secretion of inflammatory cytokines, and downstream Th1 and Th17 responses	[95]

BMDCs, bone-marrow-derived dendritic cells; CIA, collagen-induced arthritis; CII, collagen type II; IDO, indoleamine-2,3-dioxygenase; N.A., not available.

## Data Availability

The data that support the findings of this study are available upon request from the corresponding author (C.-C.L.).
